# Heterogeneity of miR-10b expression in circulating tumor cells

**DOI:** 10.1038/srep15980

**Published:** 2015-11-02

**Authors:** Christin Gasch, Prue N. Plummer, Lidija Jovanovic, Linda M. McInnes, David Wescott, Christobel M. Saunders, Andreas Schneeweiss, Markus Wallwiener, Colleen Nelson, Kevin J. Spring, Sabine Riethdorf, Erik W. Thompson, Klaus Pantel, Albert S. Mellick

**Affiliations:** 1 School of Medicine, Deakin University, Geelong, VIC, Australia; 2 Australian Prostate Cancer Research Centre-Queensland, Queensland University of Technology, Brisbane, QLD, Australia; 3 Institute of Health and Biomedical Innovation & School of Biomedical Sciences, Queensland University of Technology, Brisbane, QLD, Australia; 4 School of Surgery, The University of Western Australia, Perth, WA, Australia; 5 National Center for Tumor Diseases, Heidelberg, Germany; 6 Department of Obstetrics and Gynecology, University of Heidelberg, Heidelberg Germany; 7 Ingham Institute for Applied Medical Research, Liverpool Hospital, Liverpool, NSW, Australia; 8 Liverpool Clinical School, School of Medicine, Western Sydney University, NSW, Australia; 9 Department of Tumor Biology, University Medical Center Hamburg-Eppendorf, Hamburg, Germany; 10 St. Vincent’s Institute of Medical Research and Department of Surgery, University of Melbourne, Melbourne, Australia; 11 School of Medicine, University of New South Wales, NSW, Australia

## Abstract

Circulating tumor cells (CTCs) in the blood of cancer patients are recognized as important potential targets for future anticancer therapies. As mediators of metastatic spread, CTCs are also promising to be used as ‘liquid biopsy’ to aid clinical decision-making. Recent work has revealed potentially important genotypic and phenotypic heterogeneity within CTC populations, even within the same patient. MicroRNAs (miRNAs) are key regulators of gene expression and have emerged as potentially important diagnostic markers and targets for anti-cancer therapy. Here, we describe a robust *in situ* hybridization (*ISH*) protocol, incorporating the CellSearch^®^ CTC detection system, enabling clinical investigation of important miRNAs, such as miR-10b on a cell by cell basis. We also use this method to demonstrate heterogeneity of such as miR-10b on a cell-by-cell basis. We also use this method to demonstrate heterogeneity of miR-10b in individual CTCs from breast, prostate and colorectal cancer patients.

Circulating tumor cells (CTCs) have been demonstrated to be an independent prognostic marker of clinical outcome in patients with solid tumors^1^,^2^,^3^. Numerous assays for the enrichment and detection of CTCs have been developed and used, however the semi-automated CellSearch^®^ system (Veridex, Raritan, NJ, USA) remains the only device cleared by the Food and Drug Administration (FDA) for clinical use in metastatic breast, prostate and colon cancer^4^,^5^. To date, little is known about phenotypic characteristics of clinically significant CTCs capable of establishing metastasis at secondary sites, or the functional significance of CTC heterogeneity^6^,^7^. As a link between the primary tumor and the formation of metastasis throughout the body, CTCs are not only a promising target for future anti-metastasis therapeutic strategies, but can also be analyzed via a ‘liquid biopsy’ that can be obtained by a simple blood draw^4^,^8^. Furthermore, CTC analysis enables continuous monitoring of patient condition during the clinical course of their disease allowing early detection of recurrence and monitoring response to treatment^5^,^8^. However, the number of CTCs can be very small; as few as one CTC in 10^9^ peripheral blood mononuclear cells (PBMNCs). This rarity makes the enrichment, detection and analysis of CTCs challenging^4^,^5^,^8^. Molecular analysis of cell fractions enriched for CTCs [e.g. after epithelial cell adhesion molecule (EpCAM) enrichment] often face the problem of an over-representation of leukocytes, making data obtained by bulk CTC isolation protocols difficult to interpret^8^,^9^. Moreover, small but clinically significant subpopulations are often missed during routine analysis of primary tumor biopsies^4^,^6^,^10^,^11^, and only a few CTCs are capable of extravasating into surrounding tissue and growing as a metastasis at a secondary site^6^,^7^. These cells give important clues as to the genotypic and phenotypic changes that enable metastasis and ultimately patient death. Thus, collective analysis of individual CTCs is the most promising way of overcoming these critical issues and improving the utility of CTC analysis.

MiRNAs function as post-transcriptional regulators of gene expression and mediate a range of pathways linked to disease and development^12^,^13^. Several miRNAs, such as miR-10b have been directly linked to the epithelial-mesenchymal transition (EMT)^12^,^13^,^14^ and metastasis in breast and prostate cancer^15^,^16^,^17^,^18^. Furthermore, increased expression of miR-10b has been demonstrated in glioma, as well as metastatic pancreatic cancers^19^,^20^. Notably, increased miR-10b blood serum levels have been described in gastric cancer, non-small lung cancer and metastatic breast cancer^18^,^21^,^22^. However, as there is no association between miR-10b levels in the primary tumor and metastasis the link between miR-10b and metastasis remains controversial. It has been suggested that miR-10b may have a biological effect on a few cells at the leading edge of the tumor, or a small number of cancer cells shed into the blood^23^.

For analysis of cell-based miRNA expression *ISH* protocols using locked-nucleic acid (LNA) probes have been developed and applied to cell preparations, fresh frozen tissue and paraffin embedded tissues^24^,^25^,^26^. To enable the detection of miR-10b in distinct cell types present in a complex cellular environment such as CTCs in peripheral blood we have adapted current *ISH* protocols, combined with antibody based immunofluorescence (IF), to be used with the CellSearch^®^ CTC detection system (Fig. 1). Using this protocol we demonstrate miR-10b heterogeneity in CTCs isolated from the blood of metastatic cancer patients for the first time.

## Results

### MiR-10b expression in cancer cell lines

To identify suitable cells for method development and evaluation, commercially available breast cancer (MCF-7 and MDA-MB-231) and colorectal cancer (HCT-116 and SW480) cells were analyzed for miR-10b expression by *ISH* (Fig. 2). MiR-10b expression was detected in all cell lines. Notably, a slightly increased expression of miR-10b could be detected in the highly metastatic MDA-MB-231 cells and SW480 cells compared to less metastatic MCF-7 and HCT-116 cells, respectively.

### Spike experiment using MCF-7 and MDA-231 cells

To adapt the *ISH* procedure for analyzing CTCs detected in the blood of cancer patients with the CellSearch^®^ system, spiking experiments using blood of a healthy volunteer donor were performed. MDA-MB-231 and MCF-7 cells were spiked into 7.5 ml of blood from the healthy donor (for miR-10b probe). Additionally, two blood samples were spiked with MCF-7 cells to be used as positive (U6 probe) and negative control (scrambled probe). All samples were processed with the CellSearch^®^ system, screened with the CellTracks^®^ Analyzer to identify CK^+^ DAPI^+^ CD45^−^ tumor cells and fixed in the CellSearch^®^ cartridge. The combined *ISH*/IF protocol was applied using either miR-10b, U6-positive or scrambled-negative probes with IF staining using antibodies from the CellSearch^®^ Epithelial Cell Kit (Veridex). CTC image analysis was performed directly in the CellSearch^®^ cartridge using IF microscopy. MCF-7 cells and MDA-MB-231 cells could be detected and analyzed for miR-10b expression. As observed in previous cell line experiments MDA-MB-231 cells revealed a high miR-10b expression in comparison to the moderate miR-10b expression in MCF-7 cells (Fig. 3). No signal was detected with the scrambled-negative control probe, and strong positive signal was observed with the U6-positive probe. Cell loss during re-fixation and *ISH* procedure was found to be minimal (~1%) for freshly processed samples (within 48 h after fixation) determined by re-enumeration of spiked MCF-7 cells after *ISH* analysis (134 of 135 cells detected with the CellSearch^®^).

### MiR-10b heterogeneity in CTCs from cancer patients

To investigate miR-10b expression in CTCs in the blood of cancer patients, cells collected from 11 metastatic cancer patients (eight breast cancer, two prostate cancer and one colorectal cancer patient), were analyzed using the combined *ISH*/IF *in cartridge* protocol. MiR-10b positive (miR-10b^+^ ) as well as miR-10b negative (miR-10b^−^) CTCs could be detected in 10 of 11 patients (Figs 4 and 5a). One breast cancer patient (Breast 2) presented with 19 miR-10b^+^ CTCs only. The abundance of miR-10b^+^ CTCs in the analyzed CTC population of these 11 patients was 67% in the colorectal cancer patient, 40% and 20% in the prostate cancer and ranged from 20-94% (mean: 67%) in the breast cancer patients (Fig. 5a).

For semi-quantitative analysis of miR-10b expression in individual CTCs we established a miR-10b immunoscoring (Fig. 5b). Except for one breast cancer patient (Breast 2) with only miR10b^+^ CTCs, expression levels among CTC populations varied from ‘negative (0)’ to ‘strong (+3)’ in all patients (Supplementary Table S1).

Cell losses during the fixation and *ISH* procedure were exemplarily determined by re-enumeration of CTCs from five patients that initially presented with a total number of less than 100 CTCs. In these samples an average cell loss (±SEM) of 24.9 ± 7.6% (range: 0–55.8%), varying with length of storage prior *ISH* analysis and sample quality, was observed.

The application of our combined *ISH*/IF protocol for analyzing CTCs proved to be a robust method which revealed a heterogeneous distribution of miR-10b expression among CTCs from the same patient, across three different epithelial tumor types.

## Discussion

In this study, we describe a novel, robust combined *ISH* and IF protocol that enables expression analysis of microRNAs in definite cell types, such as CTCs. Using tumor cell line spiking experiments we adapted the protocol to be used on CTCs after being detected with the CellSearch^®^ CTC detection system. As a proof-of-principle the expression of miR-10b was investigated in 511 CTCs isolated from metastatic cancer patient with different epithelial tumor types (breast, colorectal and prostate cancer) revealing a heterogeneous miR-10b expression pattern in the CTC populations.

For method development we performed *ISH* on MCF-7 and MDA-MB-231 breast and HCT-116 and SW480 colon cell line cell preparations, detecting moderate but clear miR-10b expression in MCF-7 and HCT-116 and a high expression in MDA-MB-231 and SW480 cells. This expression pattern is consistent with previous miRNA expression studies in cancer cell lines using qPCR and array-based technologies^27^.

Though various *ISH* protocols for the analysis of messenger RNA (mRNA) have been described previously^28^,^29^,^30^ the analysis of miRNA expression is less common and requires specified adaptions^24^,^25^,^26^. In particular, the use of locked-nucleic-acid incorporated DNA probes enhances the hybridization efficiency and thus improves the detection of small miRNA molecules^31^. Several recent methods, have adapted traditional *ISH* for the analysis of miRNAs^24^,^25^,^26^. These protocols generally use LNA probes in combination with single spectrum (colorimetric or fluorescence) detection technologies, applied to formalin fixed paraffin embedded (FFPE) or fresh frozen tissues^25^,^26^ or preparations of cultured cells^28^. By combining *ISH* with IF our technique enables the analysis of specific cell subpopulations in heterogeneous tissue or cell preparation (e.g. CTCs surrounded by normal blood cells). Unlike other *ISH* protocols^24^,^25^,^29^, our protocol includes a short hybridization step only, enabling the procedure to be performed in one day. Moreover, the inherent stability across the steps allows periods of convenient storage and shipping between different sites of analysis.

Based on the heterogeneity of CTCs described previously^4^,^11^ and their prominent role in the metastasis, molecular characterisation of individual CTCs is an emerging research field. Variations in the expression of specific proteins, as well as a heterogeneous distribution of gene amplifications/mutations have been observed in several studies. For example, human epidermal growth factor receptor 2 (HER2) and epidermal growth factor receptor (EGFR) expression patterns were analyzed in CTCs detected with the CellSearch^®^ system in metastatic breast and colon cancer patients respectively^4^,^32^. Genotypic heterogeneity regarding gene mutations (e.g. in p53, KRAS, PIK3CA) and gene amplifications (e.g. EGFR, HER2, leucine-rich g-protein receptor 5, Aurora A kinase and androgen receptor) could be observed in individual CTCs from colon^4^,^11^, breast^6^ and prostate cancer patients^33^. Recently, Raemskoeld *et al.* established a protocol enabling full-length mRNA sequencing of single cells and performed mRNA expression profiling in single CTCs from a melanoma patient^34^. Our protocol is the first to enable the down-stream analysis of individual CTCs for microRNA expression. The only other study addressing microRNA analysis in CTCs of Sieuwerts *et al.* was performed on bulk CTCs after being processed using the CellSearch^®^ Profile Kit (Veridex) and procedure^9^. Unlike the CellSearch^®^ Epithelial Cell Kit this procedure includes the EpCAM enrichment only, resulting in an EpCAM-enriched cell fraction containing all CTCs and numerous remaining leukocytes. Though demonstrating the feasibility of CTC-associated microRNA profiling in a leukocyte-rich background, information regarding the distribution of miRNA expression in individual CTCs and also about the localization of the miRNA expression are not provided.

Using our technique we detected heterogeneous miR-10b expression in CTCs from patients with metastatic breast, prostate and colon cancer, respectively. MiR-10b expression has been described by Ma *et al.* and others to be linked to EMT and metastasis in breast cancer^15^,^16^,^18^. First studies also emphasize a role of miR-10b in colorectal and prostate cancer malignancy^17^,^23^,^35^,^36^. However, work from Gee *et al.* could not confirm the correlation of miR-10b expression in human breast tumors with metastasis and progression^23^, suggesting that only small highly malignant subpopulations of the primary tumor to be miR-10b positive. In this context, the detection of miR-10b^+^ CTCs, as putative founders of metastatic outgrowth, is an important finding that emphasizes the importance of miR-10b in cancer malignancy and provides avenues for further research in this field.

## Conclusion

Our protocol incorporates *ISH* with fluorescent antibody detection that allows miRNA analysis of well-defined cell populations, such as CTCs in blood of cancer patients. This has the potential to be used for various other applications such as miRNA-profiling of endothelial or immune response cells in tissue sections. The protocol was adapted for microRNA analysis of individual CTCs detected from the blood of cancer patients with the CellSearch^®^ CTC detection system. Detecting CTCs with varying miR-10b expression and the crucial role of miR-10b in metastasis and disease progression points out the potential of our *ISH* protocol to be useful for identifying and studying clinically relevant CTC subpopulations.

## Material and Methods

### Cell lines

The human mammary carcinoma cell lines MCF-7 and MDA-MB-231 as well as colorectal cancer cells HCT-116 and SW480 were obtained from the American Type Culture Collection (ATCC) (Manassas, VA, USA). MCF-7 and MDA-MB-231 cells were maintained in DMEM, HCT-116 and SW480 cells in RPMI medium (5% CO_2_, 37 °C). Cell medium was supplemented with 4.5 g/L glucose, 10% fetal bovine serum (FBS), 2 mM L-glutamine (Lonza, Basel, Switzerland), as well as 10 U/mL penicillin, and 10 μg/mL streptomycin (Life Technologies, Carlsbad, CA, USA). For *ISH* analysis cells were either grown on chamber slides (Thermo Fisher Scientific, Waltham, MA, USA) or spiked into 7.5 mL of blood from a healthy donor that was subjected to the CellSearch^®^ system (Veridex, Raritan, NJ, USA).

### Patient samples

Blood from patients diagnosed with either metastatic breast, prostate cancer or colorectal cancer was collected at the Princess Alexandra Hospital, QLD, Australia, Royal Perth Hospital, WA, Australia, St John of God Subiaco Hospital, WA, Australia, or the University Hospital Heidelberg, Germany under protocols reviewed and approved by the respective Human Research Ethics Committees. For spiking experiments blood from a healthy volunteer was used. Written informed consent was obtained from all patients and the healthy volunteer. Patients were chosen based on their clinical diagnosis of late stage metastatic disease.

For CellSearch^®^ analysis at least 7.5 mL of blood were drawn into a CellSave^®^ tube (Veridex) and stored at ambient temperature until processing (within 96 h after blood sampling).

### Locked nucleic acid (LNA) probes

*ISH* was performed using 5′ and double digoxigenin (DIG) conjugated miCURY LNA detection probes from Exiqon (Vedbæk, Denmark): (i) a U6-small nuclear RNA (positive) probe: CACGAATTTGCGTGTCATCCTT; melting temperature (T_m_): 84 °C; (ii) a scrambled (negative) probe: GTGTAACACGTCTATACGCCCA; T_m_:87 °C; and (iii) a specific anti-miR-10b probe: CACAAATTCGGTTCTACAGGGTA; T_m_:83 °C. For hybridization the following final concentrations of probe were used: U6-positive (10 nM), scrambled-negative (40 nM) and miR-10b (40 nM).

### *ISH* analysis of cancer cell lines

*ISH* analysis of cancer cell line cells was conducted to validate miR-10b expression levels. Cells grown on chamber slides were prepared for analysis by washing: once with 0.1% Triton X-100, PBS (10 min), and twice with PBS (10 min). They were then fixed (4% PFA, 5 min) and washed twice (PBS, 10 min) before blocking in hybridization buffer (50% formamide, 5 × SSC, 1 × Denhardt’s solution, 0.5 mg/mL yeast tRNA, 0.5 mg/mL salmon sperm DNA) at 50 °C (10 min). Following blocking the buffer was replaced with hybridization buffer containing DIG labeled LNA probe, and the slides left to incubate (50 °C, 45 min). The slides were then subjected to three stringency wash steps: (i) twice with 0.1 × SSC (52 °C, 10 min); (ii) once with 2 × SSC (5 min); and (iii) three times with TN Buffer (0.1 M Tris-HCl pH7.5; 0.15 M NaCl) (3 min). Prior to antibody staining, the slides were incubated in antibody blocking buffer (1% blocking reagent, Roche Diagnostics, 10% FCS, 0.15 M NaCl, 0.1 M Tris-HCL pH 7.5) for 30 min.

For probe detection cells were incubated in 1:400 diluted (in blocking buffer) anti-DIG-fluorescein (FITC)-Fab fragments (Roche Diagnostics, Basel, Switzerland) for 3 h. The slides were then washed: once with TNT buffer (1% Triton X-100 in TN-buffer, 5 min), and three times with TN buffer (3 min each). Cell nuclei were visualized using DAPI staining solution (1 ng/μL DAPI, PBS, 5 min). Cells were then washed: (i) once with 0.1% Triton X-100/PBS (5 min); (ii) twice with PBS (10 min); and (iii) twice PBS (20 min). The slides were mounted in Prolong^®^ Gold Antifade (Life Technologies, Carlsbad, California, USA). In this way slides can be stored at 4 °C for 2–4 days prior to microscopic analysis. The entire procedure was implemented in an RNAse-free working environment, with RNAse-free reagents. Unless otherwise stated steps were conducted at room temperature.

### CTC detection using the CellSearch^®^ system

CTC enrichment and detection with the semi-automated CellSearch^®^ system using the Circulating Epithelial Kit (Veridex) was performed as described previously^1^,^4^,^37^. Blood (7.5 mL) from cancer patients, or blood from the healthy volunteer spiked with cancer cell line cells (MCF-7 and MDA-MB-231) was mixed with dilution buffer (6.5 mL) and centrifuged at 800 g (10 min) prior to loading the sample into the CellSearch^®^ Autoprep system (Veridex). CTCs were immunomagnetically enriched using a ferrofluid-conjugated anti-EpCAM antibody (Veridex); and stained with 4′,6-Diamidin-2-phenylindol (DAPI), phycoerythrin (PE) conjugated anti-cytokeratin (CK) antibody and a Allophycocyanin (APC) conjugated anti-CD45 antibody as included in the Circulating Epithelial Cell Kit.

At the end of the run the enriched cell fraction (final sample volume approx. 350 μL) was collected in the CellSearch^®^ cartridge placed in a MagNest (Veridex) to direct EpCAM^+^ cells into a focal plane for microscopic analysis by the CellTracks^®^ Analyzer (Veridex). CTCs were identified as CK^+^, DAPI^+^ and CD45^−^ and by morphology (i.e. round to oval shape, high nucleus-to-cytoplasm ratio). The cartridge was not removed from the MagNest and the assembly was stored at 4 **°**C until fixation (within 24 h).

### Cell fixation in the CellSearch^®^ cartridge

To prepare cells for *ISH* analysis they were fixed directly in the CellSearch^**®**^ cartridge^38^. The buffer in the cartridge was removed and replaced with 250 μL methanol:glacial acetic acid (3:1) solution. This was removed after 2 min, and replaced with another 250 μL methanol:glacial acetic acid (3:1) solution. After another 2 min all liquid was carefully removed from the cartridge before it was dried using compressed air (10 min). The cartridge was removed from the MagNest and stored at 4 **°**C for up to three days or at −20 **°**C for long-term storage prior to *ISH* analysis. All pipetting steps were performed with Mμltiflex pipette tips (PEQLAB, Erlangen, Germany).

### Combined *ISH* and IF analysis in CellSearch^®^ cartridge

**Figure 1:**
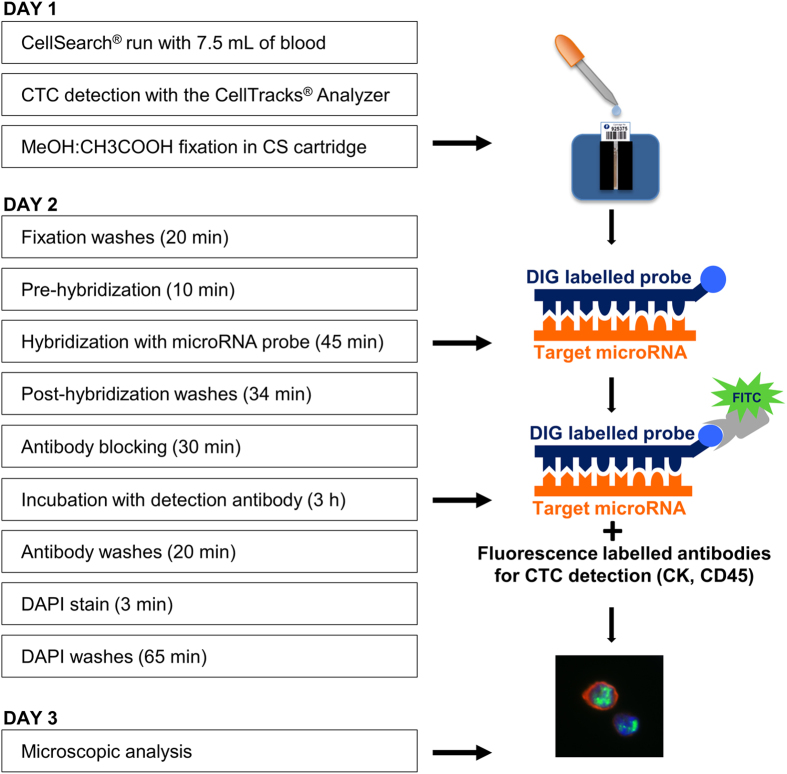
Schematic representation of the combined ISH/IF procedure. The three day stepwise protocol for analysis of CTCs detected with the CellSearch^®^ system. CTC enrichment and staining initially occurs using the CellSearch^®^. CTCs are then enumerated using the CellTracks^®^ Analyzer and fixed in the CellSearch^®^ cartridge. The *ISH*/IHC protocol using LNA probes proceeds *in cartridge*, followed by manual visualization of miR expression in CTCs on a cell-by-cell basis. DIG labelled probes detected with FITC labelled anti-DIG antibody allowed visualisation and localization of miR expression in cells, which are also re-labelled with cell specific, CTC identifying antibodies from the Circulating Epithelial Cell Kit (Veridex). CTCs can then be labelled as either miR^+^, CK^+^, DAPI^+^ and CD45^−^; or miR^−^, CK^+^, DAPI^+^ and CD45^−^, in a semi-quantitative way.

**Figure 2:**
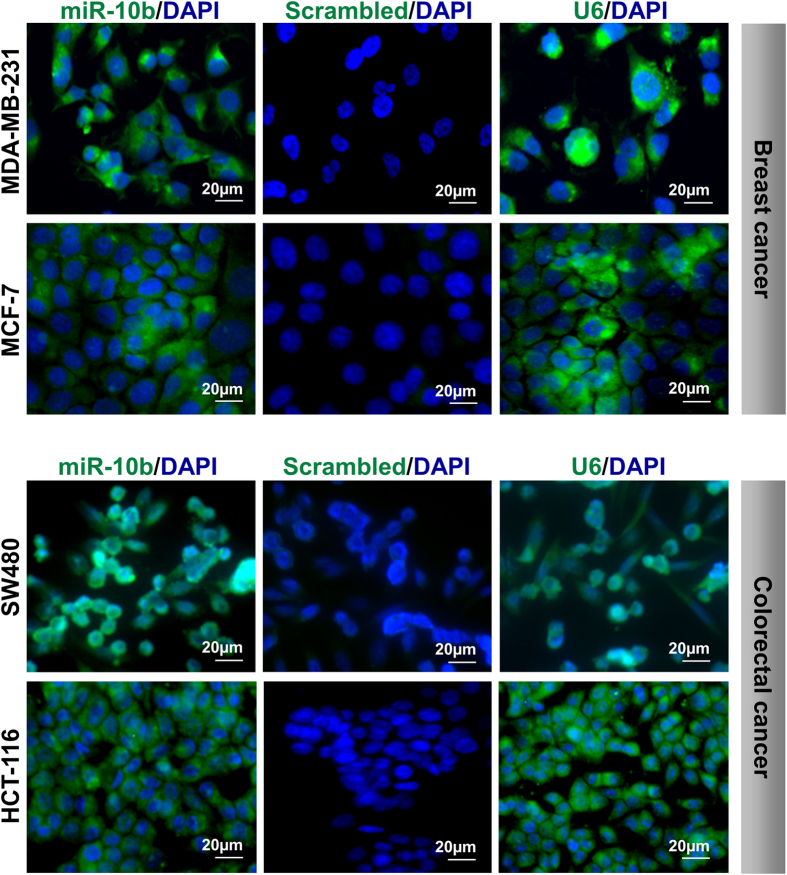
MiR-10b expression analysis in breast and colorectal cancer cell lines by ISH. Higher expressions levels of miR-10b in MDA-MB-231 cells compared with MCF-7 breast cancer cells and in SW480 compared with HCT-116 colorectal cancer cells. Cells were stained with miR-10b as well as scrambled=negative and U6-positive control probes. Nuclei were visualized with DAPI (blue). Resolution 40×. Scale bars, 20 μM.

**Figure 3:**
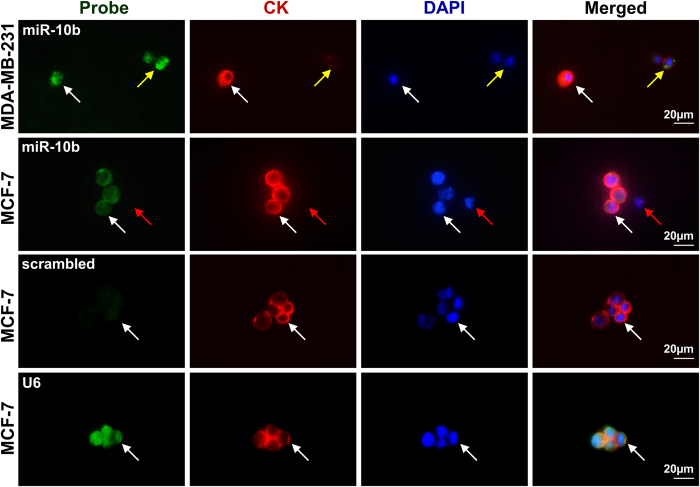
MiR-10b ISH analysis of breast cancer cell lines captured by the CellSearch® system. MiR-10b^+^ CK^+^ DAPI^+^ MCF-7 and MDA-MB-231 cells (white arrows) spiked into blood, captured by the CellSearch^®^ system and identified by image analysis. Also shown are miR-10b^−^ CK^−^ DAPI^+^ (red arrows) and miR-10b^+^ CK^−^ DAPI^+^ (yellow arrows) cells detected by IF microscopy. Also shown are the results U6-positive and scrambled-negative control probe analysis, respectively. Nuclei were visualized with DAPI. Resolution 40×. Scale bars, 20 μM.

**Figure 4:**
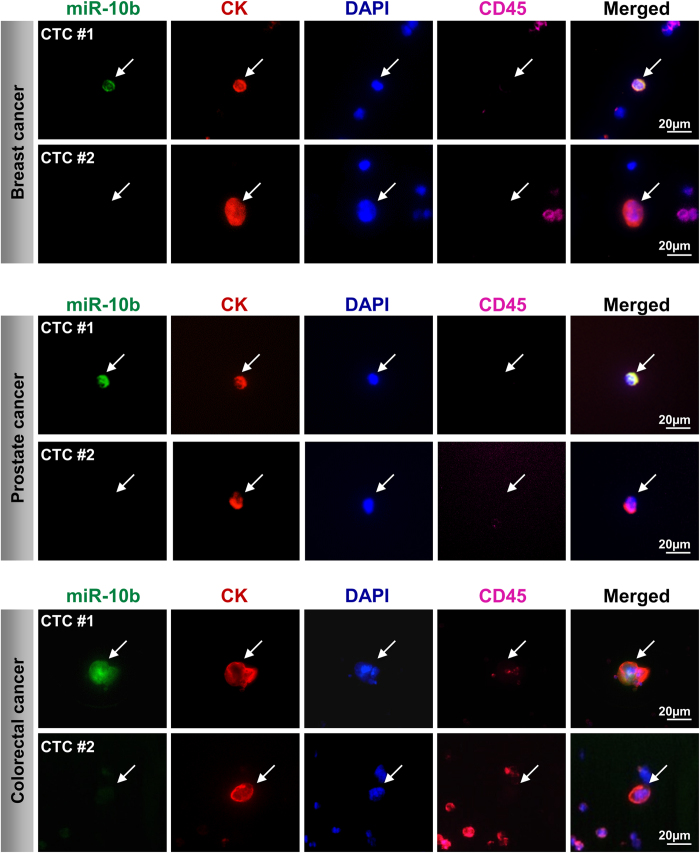
MiR-10b expression analysis of CTCs from metastatic cancer patients by *ISH*. Shown, are miR-10b^+^ CK^+^ DAPI^+^ CD45^−^ CTCs and miR-10b^−^ CK^+^ DAPI^+^ CD45^−^ CTCs (white arrows) captured from the blood of metastatic breast, prostate and colorectal cancer patients. Nuclei were visualized with DAPI. Resolution 40×. Scale bars, 20μM.

**Figure 5:**
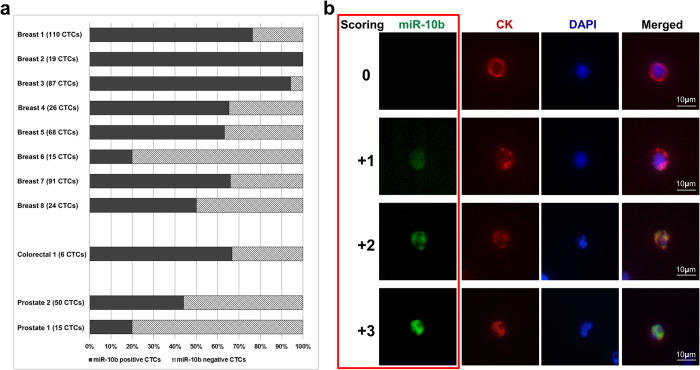
Heterogeneity of miR-10b expression in CTCs from metastatic cancer patients. (**a**) Distribution of miR-10b^+^ and miR-10b^−^ CTCs in the analyzed CTC populations from eight breast, one colorectal and two prostate cancer patients. (**b**) Immunoscoring based on miR-10b signal intensity for semi-quantitative analysis of miR-10b expression. Shown are four CTCs from one prostate cancer patient: one miR-10b^−^ (scoring: 0), and one with weak (+1), moderate (+2) and strong (+3) miR-10b expression. Nuclei were visualized with DAPI. Resolution 20×, Scale bars, 10 μM.

Combined *ISH* and IF analysis was conducted directly in CellSearch^®^ cartridges previously subjected to fixation (Fig. 1). Cartridges were filled with 0.1% Triton X-100, PBS. After 10 min it was replaced with PBS and left for another 10 min. Hybridization buffer (350 μL) for blocking was added, and the cartridge left to incubate (30 min, 50 **°**C). Hybridization buffer containing LNA probes was then added, and the cartridge left to incubate for a further 45 min (50 **°**C). After hybridization cartridges were subjected to three stringency washes: (i) twice with 0.1× SSC (10 min, 52 °C); (ii) once with 2× SSC (5 min); and (iii) three times with TN Buffer (3 min each).

For LNA probe detection, and re-staining of antigens (CK and CD45), cells were blocked with antibody blocking buffer (30 min) and incubated (3 h) with 1:400 dilution of anti-DIG Fab fragments (Roche Diagnostics), as well as 1:6 dilution of CellSearch^®^ antibody mixture (Cell Search Epithelial Cell Kit, Veridex,). The cartridge was then washed: (i) once with TNT buffer (5 min); and (ii) three times with TN buffer (5 min each). Cell nuclei were visualised with DAPI (1 ng/μL in PBS, 5 min) and cells washed again: (i) once in 0.1% Triton X-100, PBS (10 min); (ii) four times with PBS (10 min). Cartridges were filled with PBS and stored at 4 °C for up to two days prior microscopic analysis. All pipetting steps were performed with Mμltiflex pipette tips (PEQLAB). Unless otherwise stated steps were conducted at room temperature.

### Microscopic analysis

Imaging of cells grown on slides and of cells in the CellSearch^®^ cartridges was performed on either a Zeiss LSM5 Live (Carl Zeiss, Jena, Germany) or an Olympus IX71 inverted fluorescence microscope (Olympus, Tokio, Japan). Image analysis was therefore conducted using the AxioVison Rel. 4.8 Imaging software (Carl Zeiss) or OlyVia ver. 2.8. Imaging software (Olympus), respectively.

## Additional Information

**How to cite this article**: Gasch, C. *et al.* Heterogeneity of miR-10b expression in circulating tumor cells. *Sci. Rep.* **5**, 15980; doi: 10.1038/srep15980 (2015).

## Supplementary Material

Supplementary Information
