# Supplementary material for: Heterogeneity of miR-10b expression in circulating tumor cells
Source: Sci Rep. 2015 Nov 2;5:15980. doi: 10.1038/srep15980 (PMC4629160; doi:10.1038/srep15980)
Supplement: Supplementary Information [file srep15980-s1.doc]

**Heterogeneity of miR-10b expression in circulating tumor cells**

Christin Gasch^1^**^¶^**, Prue N. Plummer^1^**^¶^**, Lidija Jovanovic^2,3^, Linda M. McInnes^4^, David Wescott^1^, Christobel M. Saunders^4^, Andreas Schneeweiss^5,6^, Markus Wallwiener^5,6^, Colleen Nelson^2,3^, Kevin J. Spring^7^, Sabine Riethdorf^8^, Erik W. Thompson^3,9^, Klaus Pantel^8^, Albert S. Mellick^1*^.

1. School of Medicine, Deakin University, Geelong Waurn Ponds Campus, Geelong, VIC, Australia.

2. Australian Prostate Cancer Research Centre-Queensland, Translational Research Institute, Brisbane, QLD, Australia.

3. Institute of Health and Biomedical Innovation & School of Biomedical Sciences, Queensland University of Technology, Brisbane, QLD

4. School of Surgery, The University of Western Australia, Perth, WA, Australia.

5. National Center for Tumor Diseases, Heidelberg, Germany

6. Department of Obstetrics and Gynecology, University of Heidelberg, Germany

7. Ingham Institute, Liverpool Hospital, Liverpool Clinical School, University of Western Sydney, NSW, Australia.

8. Department of Tumor Biology, University Medical Center Hamburg-Eppendorf, Hamburg, Germany.

9. St. Vincent’s Institute, Melbourne and University of Melbourne Department of Surgery, St. Vincent’s Hospital, Melbourne, Australia.

*Corresponding author

Email: albert.mellick@deakin.edu.au (ASM)

**^¶^**These authors contributed equally to this work.

**Supplementary**

**Table S1. MiR-10b immunoscoring of CTCs.**

| **Patient** | **Total number of**  **analyzed CTCs** | **MiR-10b immunoscoring of CTC** | | | |
| --- | --- | --- | --- | --- | --- |
|  |  | **0** | **+1** | **+2** | **+3** |
| **Breast cancer patients** | | | | | |
| 1 | 110 | 26 | 15 | 28 | 41 |
| 2 | 19 | 0 | 4 | 4 | 11 |
| 3 | 87 | 5 | 27 | 25 | 30 |
| 4 | 26 | 9 | 11 | 1 | 5 |
| 5 | 68 | 25 | 6 | 17 | 20 |
| 6 | 15 | 12 | 1 | 0 | 2 |
| 7 | 91 | 31 | 28 | 22 | 10 |
| 8 | 24 | 12 | 10 | 2 | 0 |
| **Prostate cancer patients** | | | | | |
| 1 | 50 | 28 | 4 | 8 | 10 |
| 2 | 15 | 12 | 0 | 1 | 2 |
| **Colorectal cancer patient** | | | | | |
| 1 | 6 | 2 | 2 | 2 | 0 |
